# Construction of a Lectin–Glycan Interaction Network from Enterohemorrhagic *Escherichia coli* Strains by Multi-omics Analysis

**DOI:** 10.3390/ijms21082681

**Published:** 2020-04-12

**Authors:** Seung-Hak Cho, Kang Mo Lee, Cheorl-Ho Kim, Sung Soon Kim

**Affiliations:** 1 Division of Bacterial Disease Research, Center for Infectious Disease Research, Korea National Institute of Health, Cheongju, Chungchungbuk-do 28160, Korea; skcho38@korea.kr (S.-H.C.); km88lee@korea.kr (K.M.L.); 2 Glycobiology Unit, Department of Biological Science, Sungkyunkwan University and Samsung Advanced Institute for Health Science and Technology (SAIHST), Suwon, Gyeonggi-do 16419, Korea

**Keywords:** enterohemorrhagic *Escherichia coli* (EHEC), lectin–glycan interactions (LGIs), multi-omics analysis, lectin-like adhesins, outer membrane-embedded proteins

## Abstract

Enterohemorrhagic *Escherichia coli* (EHEC) causes hemorrhagic colitis and hemolytic uremic syndrome. EHEC infection begins with bacterial adherence to the host intestine via lectin-like adhesins that bind to the intestinal wall. However, EHEC-related lectin–glycan interactions (LGIs) remain unknown. Here, we conducted a genome-wide investigation of putative adhesins to construct an LGI network. We performed microarray-based transcriptomic and proteomic analyses with *E. coli* EDL933. Using PSORTb-based analysis, potential outer-membrane-embedded adhesins were predicted from the annotated genes of 318 strains. Predicted proteins were classified using TMHMM v2.0, SignalP v5.0, and LipoP v1.0. Functional and protein–protein interaction analyses were performed using InterProScan and String databases, respectively. Structural information of lectin candidate proteins was predicted using Iterative Threading ASSEmbly Refinement (I-TASSER) and Spatial Epitope Prediction of Protein Antigens (SEPPA) tools based on 3D structure and B-cell epitopes. Pathway analysis returned 42,227 Gene Ontology terms; we then selected 2585 lectin candidate proteins by multi-omics analysis and performed homology modeling and B-cell epitope analysis. We predicted a total of 24,400 outer-membrane-embedded proteins from the genome of 318 strains and integrated multi-omics information into the genomic information of the proteins. Our integrated multi-omics data will provide a useful resource for the construction of LGI networks of *E. coli*.

## 1. Introduction

The gastrointestinal tract in humans is covered by mucosal epithelial cells, providing a barrier to defend against microbial attack. The mucosal barrier is coated by the glycocalyx, an extracellular mesh of carbohydrate-rich molecules bound to cell membranes or secreted by cells into the external milieu [1]. The thickness of mucosal surfaces ranges from 300 µm in the stomach to 700 µm in the intestine [2,3]. Many defensive compounds are secreted into the mucosal fluid and form a physical barrier [4]. The commensal microbiota lives in the outer layer of the mucosal barrier and uses mucin glycans as nutrients made available by glycan-degrading enzymes [5].

During infection, enteric bacterial pathogens, including enterohemorrhagic *Escherichia coli* (EHEC), first interact with gut microbiota that are resistant to enteric pathogens by competing for resources and through training mucosal immune cells [6]. Next, they adhere to the host intestine through the binding of lectin-like adhesins to receptors of the host, including glycans [7]. These interactions involve specific binding processes by glycosylated molecules, such as glycoprotein mucin, which can play a role in colonization and disease [8,9,10]. Four mucins, MUC2, MUC5AC, MUC5B, and MUC6, constitute the mucosal barrier in the human gastrointestinal tract [11]. These glycans can be used as nutritional sources by enteric bacterial pathogens during infection (e.g., MUC2) [12].

Bacterial pathogenesis is caused by lectin-like virulence proteins that can be considered as drug targets and vaccine components. Bacterial adhesins are lectin proteins with host-cell adhesion potential and diverse structural architectures [13]. They include capsules, vesicles, pili, fimbriae, and enzymes. They recognize host cell surface receptor proteins and contribute to several biological events, including cross-membrane trafficking and invasion. Eventually, they cause pathological toxicities such as inflammation. Some adhesions are specific to mannose in immune activation, and therefore mannose supplementation and receptor blocking may disrupt the adhesin–receptor interaction. For example, the *Acinetobacter baumannii* glycoprotein PilA binds to selectins and CEACAMs of host cells [14]. Other lectin-like proteins are surface antigen 20 (CS20) and fimbriae (FimH, Yad) protein SfaS in *Escherichia coli* [15]; surface-adhesin protein E in *Hemophilus influenzae* [16]; autotransporter adhesin in *Neisseria meningitidis* [17]; ShdA, MisL, Sad, and BapA in *Salmonella enterica* serovar *Enteritidis* [18]; as well as polysaccharide intercellular adhesin (PIA) in *Staphylococcus epidermidis* [19].

EHEC is a major cause of gastrointestinal diseases such as hemorrhagic colitis and hemolytic uremic syndrome [20,21], and low infection doses cause disease development [22]. It also possesses two major Shiga toxins (Stx), designated Stx1 and Stx2, which are the major virulence factors [23].

However, the information on the lectin–glycan interaction (LGI) of EHEC is not well known. Therefore, in this study, we conducted a genome-wide investigation of putative adhesins to construct an LGI network. In addition, we selected lectin candidates by comparison with transcriptomic and proteomic data for mucin recognition in EHEC.

## 2. Results

### 2.1. Identification of Proteins That Interact with Host Mucin Using Transcriptomic and Proteomic Analysis

Little is known about the interactions between bacteria and host mucin, and how these affect colonization and pathogenicity. To investigate the effect of host mucin on EDL933 gene expression, we profiled the transcriptome of EDL933 cultured with porcine stomach mucin (0.5%). A total of 320 genes were upregulated more than twofold when EDL933 was cultured with mucin. Meanwhile, 412 genes were downregulated by mucin exposure.

On the other hand, two-dimensional (2D) gel electrophoresis was conducted to observe protein-level control of pathogenic factors by mucin. We confirmed that the presence or absence of mucin resulted in strikingly different protein patterns. Most proteins were found to have a slightly acidic or acidic pI value, with the broadest distribution of pI values between 4.0 and 5.0. In terms of molecular weight, protein weight ranged between 23 and 65 kDa.

**Figure 1:**
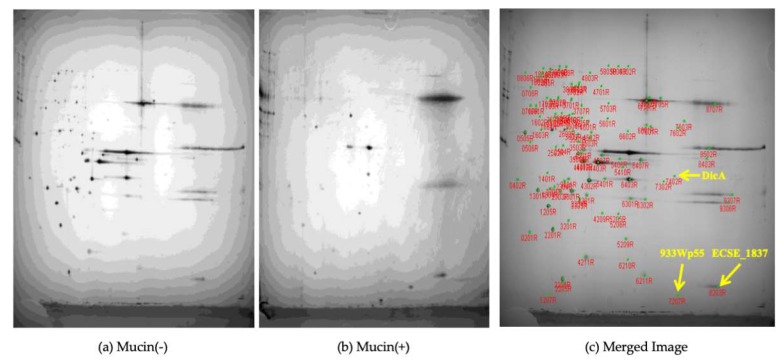
Changes in the extracellular secretion of *Escherichia coli* EDL933 proteins after mucin exposure, as detected by 2D gel electrophoresis: Secreted proteins in the absence (**a**) and presence (**b**) of mucin. (**c**) Merged image of (**a**) and (**b**). Three proteins, 933Wp55 [24], DiCA [25], and ECSE_1837 [26], which were identified in the presence of mucin are highlighted in yellow letters.

Notably, three proteins were identified only in the presence of mucin (Table S1). In contrast, 85 proteins were identified in the absence of mucin. Only 22 of the 110 candidate proteins overlapped between groups, and the remaining proteins showed a definite change in the presence or absence of mucin (Figure 1).

### 2.2. Prediction of Lectin Candidate Proteins and Protein Functional Analysis

**Figure 2:**
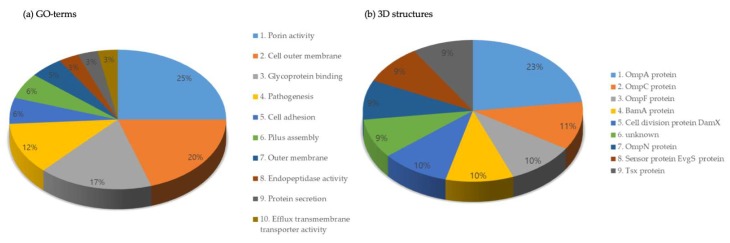
The ten most abundant proteins according to the Gene Ontology (GO) terms and 3D structures. (**a**) GO terms associated with outer-membrane-embedded proteins. (**b**) 3D structures of the outer-membrane-embedded proteins.

We predicted a total of 24,400 outer-membrane-embedded proteins from the genome of 318 strains with our lectin prediction pipeline (Table S2). We identified 227 genes according to the definition of outer-membrane-embedded protein. We assigned 24,400 genes to a total of 42,227 Gene Ontology (GO) terms. According to the GO terms, the ten most enriched pathways associated with outer-membrane-embedded proteins were porin activity, outer cell membrane, glycoprotein binding, pathogenesis, cell adhesion, pilus assembly, outer membrane, endopeptidase activity, protein secretion, and efflux transmembrane transporter activity (Figure 2a). The results of the InterProScan analysis revealed the 3D structures of 26,385 outer-membrane-embedded proteins. Among these 3D structures, the most abundant were immunoglobulins, unintegrated signature, outer membrane usher protein FimD [27], unknown, TonB-dependent receptor [28], serralysin-like metalloprotease, and outer membrane protein (Figure 2b).

### 2.3. Analysis of the Protein–Protein Interaction (PPI) of Lectin Candidate Proteins

**Figure 3:**
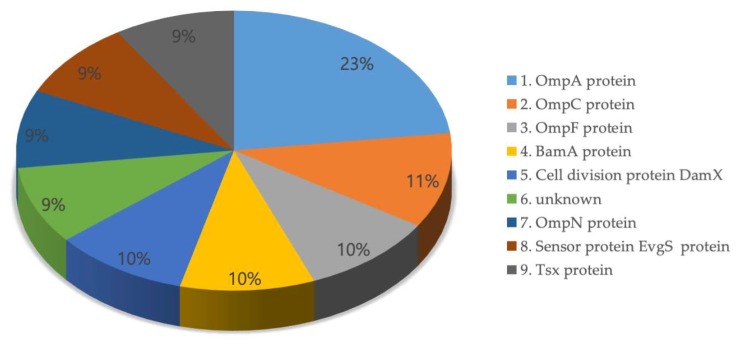
The most common interacting proteins of the outer-membrane-embedded proteins. The protein–protein interaction (PPI) information was obtained from the String database (version 11.0) [35].

**Figure 4:**
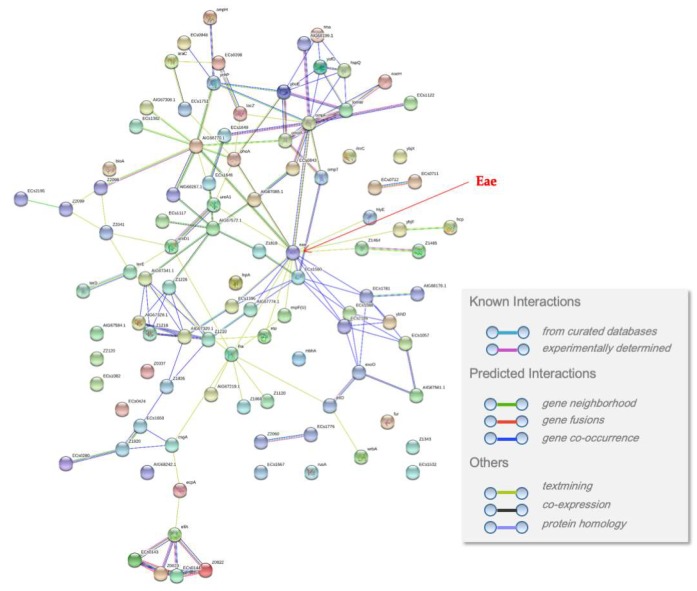
The PPI network of the predicted outer-membrane-embedded protein Eae. Eae is indicated with a red arrow. The PPI information was retrieved from the String database (version 11.0) [35].

There were 502,803 results of the PPI information of the predicted outer-membrane-embedded proteins. From the PPI information, the most common interacting proteins were OmpA [29], OmpC, OmpF [30], BamA (ß-barrel assembly machinery) [31], DamX (cell division protein) [32], OmpN, EvgS (sensor protein) [33], and Tsx (nucleoside-specific channel) [34] (Figure 3). The PPI data supported the identification of lectins among the predicted outer-membrane-embedded proteins. For instance, using the PPI information, we investigated the interactome of Eae and found that most of the interacting proteins are lectin-related (Figure 4, Table S2).

### 2.4. Homology Modeling of the 3D Structure and Epitope Prediction of Lectin Candidate Proteins

Starting with a total of 24,400 outer-membrane-embedded proteins, we selected 2585 lectin candidate proteins, belonging to five categories: adhesins, Csg, Eae, FimH, and fimbrial proteins. The 2585 lectin candidate proteins have 64 Entrez gene IDs. We performed homology modeling of the 3D structure of lectin candidate proteins using Iterative Threading ASSEmbly Refinement (I-TASSER) and obtained 63 homology models, but could not generate a homology model of CsgA as no template structure was available. B-cell epitope regions were analyzed with predicted homology models using the Spatial Epitope Prediction of Protein Antigens (SEPPA) server. The estimated B-cell epitope regions were comparatively analyzed with those of previously identified lectin-related proteins or experimentally tested lectins, in terms of ligand binding affinity.

### 2.5. Selection of Lectin Candidates by Comparison with Data of Transcriptome Expression by Mucin Recognition in EHEC

Of 172 genes, 7 genes were excluded. Transcriptional profiling revealed 425 downregulated mobility-related genes (fold change ≥2.0) in response to mucin exposure. Among the 165 genes, 5 genes were upregulated (fold change ≥2.0) by the mucin recognition signal: 2 lipoprotein genes, *rlp*B [36] and *yjb*H [37], and 3 outer membrane protein genes, *lom*P [38], *yia*D [39], and *yqh*H [39].

## 3. Discussion

To investigate the interactions between host mucin and pathogen proteins, we performed a series of transcriptomic and proteomic analyses with the EDL933 strain.

The transcriptomic analysis revealed more upregulated genes than downregulated ones after mucin exposure. Among upregulated genes, there were a number related to intestinal adherence related genes such as Type III secretion system-related genes, curli-related genes, and fimbrial genes. Interestingly, however, eight genes related to flagella biosynthesis were downregulated by exposure to mucin. Our proteomic analysis showed different protein patterns in the presence or absence of mucin. As mentioned in the results, only three proteins, 933Wp55 [24], DiCA [25], and ECSE_1837 [26], were identified exclusively in the presence of mucin, whereas 85 proteins were identified in the absence of mucin (Figure 1, Table S1). The role of the three proteins in the interactions between host mucin and pathogen proteins are unknown. The proteome analysis suggests that mucin acts as a primary barrier of the intestinal tract and is markedly reduced in extracellularly secreted proteins, which supposedly contain pathogenic intestinal hemorrhagic *E. coli*-associated factors. However, secreted pathogenic proteins may bind to individual domains of specific mucins, especially glycoproteins, and may be degraded.

This phenomenon demonstrates that, although more proteins are seemingly involved in the internal synthesis, *E. coli* protein secretion is abolished by mucin. Interestingly, the expression of the flagella capping protein (a protein related to flagellum construction) was reduced, which may be consistent with the results of transcriptome analysis, showing reduced *E. coli* motility-related factors during early infection. A former study showed that EDL933 flagella-related genes were repressed, and the motility was inhibited by mucin [40].

**Figure 5:**
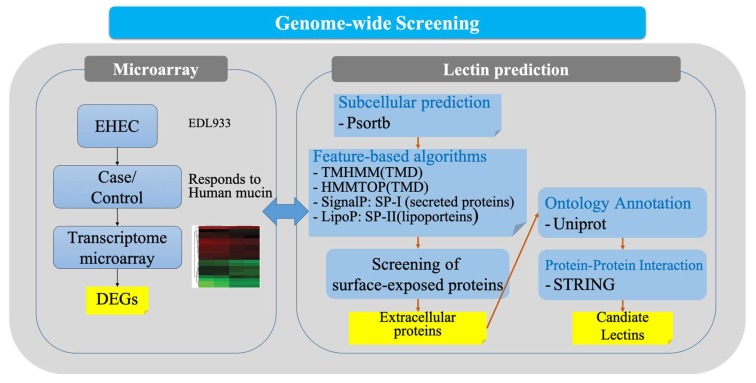
A two-step approach for lectin candidate prediction.

To predict lectin candidate proteins, we developed a prediction pipeline to analyze genes encoding outer-membrane-embedded proteins [41] for *E. coli* (Figure 5) and predicted 24,400 outer membrane-embedded proteins from the genome of 318 strains (Table 1 and Table S2). From these analyses, we selected 227 genes according to the respective proteins. Among the outer-membrane-embedded proteins, several proteins were already known as adhesins, such as Eae [42], FdeC [43], and SinH [44]. Stx is the major virulence factor of EHEC strains, which are also termed Shiga toxin-producing *E. coli* (STEC). Typical STEC strains possess a 35-kb locus of enterocyte effacement (LEE) pathogenicity island containing the *eae* gene, which encodes an outer membrane protein (intimin) required for intimate attachment to epithelial cells. Meanwhile, FdeC is a broadly conserved *E. coli* adhesin that may serve as a target to treat and prevent urinary tract infections. Besides, SinH is also known as an intimin-like protein in *Salmonella enterica* subsp. enterica serovar Typhimurium str. LT2.

Additionally, we integrated structural information (domain and 3D structure) (Figure 2a,b), transcriptomic information (microarray data), and proteomic information (PPIs) ( Figure 3; Figure 4) into the genomic information of the proteins. When we analyzed the multi-omics data, we identified that proteins predicted as outer-membrane-embedded proteins participated in the interaction with lectin-related proteins. Furthermore, we selected a total of 2585 lectin candidate proteins from multi-omics analysis and performed homology modeling and B-cell epitope analysis.

As a result, our integrated multi-omics data and prediction pipeline provide a useful resource for the construction of LGI networks of *E. coli*. However, the present study has some limitations, and additional T7-phage display experiments are required to validate the putative adhesins, and to construct a more comprehensive LGI network. The LGI network will serve as a theoretical framework to guide researchers toward successful vaccine or medicine development against EHEC.

## 4. Materials and Methods

### 4.1. Strains and Culture

For transcriptomic analysis and proteome analysis, we selected a strain of EDL933 [45]. The strain was cultured on M9 minimum medium with glucose (0.4% (*v*/*v*)) and sodium bicarbonate (44 mM (*v*/*v*)), before growing in Luria-Bertani (LB) medium at 37 °C for 18 h. To investigate the effect of mucin, we supplemented the M9 minimum medium with porcine stomach mucin (0.5% (*v*/*v*)) (type III from the porcine stomach) (Sigma, Saint Louis, MO, USA). For genome-wide screening of lectin candidates, a total of 318 EHEC strains were used (Table 1). The strains include isolates from thirteen countries and a reference strain, EDL933. The EHEC strains and their epidemiological information were retrieved from the Joint Genome Institute (JGI) Genome portal. The assembly data were retrieved from the NCBI Sequence Read Archive. The isolated strains from Korea were deposited in the National Culture Collection for Pathogens (NCCP) at the Korean National Institute of Health and their genomes were already published: NCCP15738 [46], NCCP15647 [47], NCCP15658 [48], NCCP15739 [49], NCCP15655 [50], NCCP15656 [50], NCCP15736 [51], and NCCP15737 [51].

### 4.2. Transcriptomic Analysis

Bacterial RNAs from the early stationary phase culture (0.8 OD600) were extracted using the RNeasy Midi-Prep kit (Qiagen, Hilden, Germany) after growing in LB broth at 37 °C. Transcriptomic analysis was performed using OciChip™ (E-Biogen, Seoul, Korea). Image analysis was performed using GenePix Pro 6.0 (Axon Instruments, Union City, CA, USA), and data analysis was performed using GeneSpring 7.3.1 (Agilent Technologies, Santa Clara, CA, USA). We employed the locally weighted scatterplot smoothing (LOWESS) method to normalize intensities.

### 4.3. Proteomic Analysis

Bacterial protein samples were prepared using the following protocol: strains were incubated under the same conditions as in the transcriptomic analysis, and we prepared extracellular protein samples by collecting the supernatant fraction, excluding the cell pellet. The pellet and supernatant were separated using a high-speed centrifuge. To prevent the cells from contaminating the supernatant, it was filtered through a 0.22 µm syringe filter. The trichloroacetic acid solution was added to the filtered sample at 10% of the total volume, and the protein in the supernatant was concentrated at 4 °C for 18 h. The precipitated protein was collected by centrifugation, and the protein pellet was washed three to four times with acetone. The washed sample was completely dried at room temperature, and the protein lysis solution (7 M urea, 2 M thiourea) was added to suspend the sample. Protein concentration was measured using the Bradford assay.

First-dimension isoelectric focusing (IEF) was performed to separate each protein by a unique isoelectric point (pI) value. The reaction conditions of IEF were 12 h of rehydration, 1 h at 1000 V, 1 h at 2000 V, and a final 10 h at 8000 V. A total of 24 h of one-dimensional electrophoresis was performed. After the one-dimensional electrophoresis, two-dimensional electrophoresis was performed using polyacrylamide gel electrophoresis. After the electrophoresis, the gel was stained by a series of processes using a silver stain kit to identify the location of the protein band.

### 4.4. Genome-Wide Prediction of Lectin Candidate Proteins

We predicted lectin candidate proteins using a two-step approach (Figure 5). First, we performed a genome-wide screening of outer-membrane-embedded proteins. Potential adhesins in the outer membrane were predicted from annotated genes of 318 strains using the PSORTb program (version 3.0.2) [52]. The predicted outer-membrane-embedded proteins were classified by TMHMM (version 2.0) [53], SignalP (version 5.0) [54], and LipoP (version 1.0) [55].

### 4.5. Comparison of Genome-Wide Predicted Candidates with Transcriptomic Data and Mucin Recognition Sites in EHEC

After the genome-wide screening, we performed a comparison of lectin candidate proteins with transcriptome data to identify mucin recognition sites. In the microarray-based transcriptome study, we identified mobility-related genes. Mucin recognition signals downregulated genes. In this study, we compared the predicted outer-membrane-embedded proteins to the microarray study and excluded mobility-related genes. In contrast, we selected upregulated expression genes as lectin candidates.

### 4.6. Analysis of the Function of Lectin Candidate Proteins

The function of the predicted outer-membrane-embedded proteins was classified by GO analysis using InterProScan (version 5.33-72.0, EMBL-EBI, Hinxton, UK) [56]. To retrieve the GO terms, we used the “-goterms” option during the analysis. Furthermore, the domain structure of the outer-membrane-embedded proteins and 3D structure information was investigated using the InterProScan database.

### 4.7. Analysis of the PPIs of Lectin Candidate Proteins

Outer-membrane-embedded proteins interact with various molecules, such as proteins and chemicals. A proposed lectin protein may participate in the interaction with other lectin-related proteins. Therefore, we retrieved the PPI information of the homologous protein of the predicted outer-membrane-embedded proteins from the String database (version 11.0, ELIXIR, Hinxton, UK) [35]. To search homologous proteins, a BLAST search was conducted against the subset of String databases for *E. coli*. The PPI information of homologous proteins was integrated into the annotation of the predicted outer-membrane-embedded proteins.

### 4.8. Homology Modeling of the 3D Structure and Epitope Prediction of Lectin Candidate Proteins

The 3D structure of domains was investigated for lectin candidate proteins using InterProScan. However, that structural information is from reference or experimentally confirmed strains. Therefore, we performed homology modeling of the 3D structure of lectin candidate proteins using I-TASSER, version 5.1 [57] to obtain an individual structure. We used 3D structure information predicted by InterProScan as a reference for homology modeling. Following homology modeling, we analyzed B-cell epitopes from the predicted 3D structure using Spatial Epitope Prediction of Protein Antigens (SEPPA), version 2.0 [58]. The predicted B-cell epitope will be compared with the B-cell epitope region of known lectin proteins in future analyses.

## Figures and Tables

**Table 1:** Enterohemorrhagic *Escherichia coli* (EHEC) strains used for this study.

Isolation Country	Count
Argentina	6
Brazil	1
Canada	1
Denmark	2
France	1
Germany	8
Japan	3
Netherlands	128
Norway	4
South Korea	8
Sweden	2
United Kingdom	10
USA	141
Not identified	2
Reference (EDL933)	1

## Supplementary Materials

Supplementary Materials can be found at https://www.mdpi.com/1422-0067/21/8/2681/s1. **Table S1.** Proteins identified by proteome analysis in the presence or absence of mucin; **Table S2.** PPI network information of Eae from the String database (version 11.0).

## Abbreviations

EHEC	Enterohemorrhagic *Escherichia coli*
GO	Gene Ontology
IEF	isoelectric focusing
JGI	Joint Genome Institute
LB	Luria-Bertani
LEE	locus of enterocyte effacement
LGI	lectin–glycan interaction
LOWESS	locally weighted scatterplot smoothing
NCCP	National Culture Collection for Pathogens
PIA	polysaccharide intercellular adhesin
PPI	Protein–Protein Interaction
SEPPA	Spatial Epitope Prediction of Protein Antigens
STEC	Shiga-toxin-producing *Escherichia coli*
Str.	Strain
